# A Case of Statin-Associated Immune-Mediated Necrotizing Myopathy, Successfully Treated With Intravenous Immunoglobulin

**DOI:** 10.7759/cureus.16001

**Published:** 2021-06-28

**Authors:** Mishouri Paul, Prodip Paul, Dipon Dey, Syed W Moazzem, Fariya Shamrin

**Affiliations:** 1 Medicine, Interfaith Medical Center, New York City, USA; 2 Internal Medicine, Geisinger Community Medical Center, Scranton, USA; 3 Epidemiology and Public Health, ZWH Medical Care PC, Queens, USA; 4 Rheumatology, Rush Memorial Hospital, Rushville, USA; 5 Neuroscience, Indiana University, Bloomington, USA

**Keywords:** immune-mediated necrotizing myopathy, anti hmgcr antibody, intravenous immunoglobulin (ivig), statin-induced myopathy, inflammatory myopathies

## Abstract

Statins have become the commonest lipid-lowering agent worldwide and have significantly reduced morbidity and mortality associated with cardiovascular diseases. Overall, statins are very well tolerated. However, in clinical practice, a wide variety of skeletal myopathic effects have been observed, ranging from asymptomatic patients with high creatine phosphokinase (CPK) to fatal cases of acute rhabdomyolysis. Recent reports suggest that statins are associated with immune-mediated necrotizing myopathy (IMNM), a unique autoimmune myopathy. Unlike other drug reactions, this can occur months to years after initiation of statin. It is a distinctive autoimmune myopathy where symptoms persist or even progress after statin discontinuation and requires immunosuppressive therapy. The presence of anti-hydroxy-methyl-glutaryl coenzyme-A reductase (HMGCR) antibody in serum strengthens the diagnosis of statin-associated necrotizing myopathy. Here we present a case of statin-associated IMNM in a 43-year-old Caucasian female who had statin-induced progressive deterioration of proximal muscle weakness with poor response to high-dose steroids and required further immunosuppressive therapy.

## Introduction

Statins, also called hydroxy-methyl-glutaryl-coenzyme-A reductase (HMGCR) inhibitors, are generally considered safe and are well-tolerated drugs. Statins may be responsible for a wide array of musculoskeletal manifestations, such as an asymptomatic rise in creatine phosphokinase (CPK), myalgias, myositis, and rhabdomyolysis [1]. Statin therapy-associated muscle problems have been reported in approximately 10%-25% of patients treated in clinical practice [2,3].

Inflammatory myopathies are characterized by progressive proximal muscle weakness due to autoimmune-mediated damage to muscle tissue. Most well-known inflammatory myopathies associated with statins include polymyositis (PM), dermatomyositis (DM), and inclusion body myositis [4,5]. However, a novel entity of statin-associated immune-mediated necrotizing myopathy (IMNM) has been reported, which is distinct from statin-induced myalgias and inflammatory myopathies mentioned above. IMNM is considered as a rare and severe adverse effect of statins in which patients usually present with a severe symmetrical proximal myopathy associated with a significantly high serum CPK level [6]. The estimated incidence of IMNM is approximately two to three of every 100,000 patients treated with statins [7]. These autoimmune myopathies are characterized clinically by symmetric proximal muscle weakness, elevated serum CPK levels, and myopathic findings on electromyography (EMG) [8]. The presence of autoantibodies and histological examination is used to differentiate among the different subtypes of statin-induced inflammatory myopathy [4].

There are two subtypes of IMNM including anti-3-hydroxy-3-methyl-glutaryl-coenzyme A reductase (anti-HMGCR) and anti-signal recognition particle (SRP) [9]. Statins are responsible for the upregulation of HMGCR expression, the major target of autoantibodies in statin-associated IMNM. Expression of high levels of HMGCR in regenerating muscle fiber may sustain the immune response even after discontinuation of statins [10]. Specific autoantibody directed against HMGCR in patients with IMNM can help identify these patients in clinical practice and determine the need for immunosuppressive therapy. This autoimmune necrotizing myopathy, where symptoms persist or even progress after statin discontinuation, responds well after initiation of immunosuppressive therapy [5]. Here, we present a case of statin-associated IMNM in a 43-year-old female who was successfully treated with intravenous immunoglobulin (IVIG).

## Case presentation

**Figure 1:**
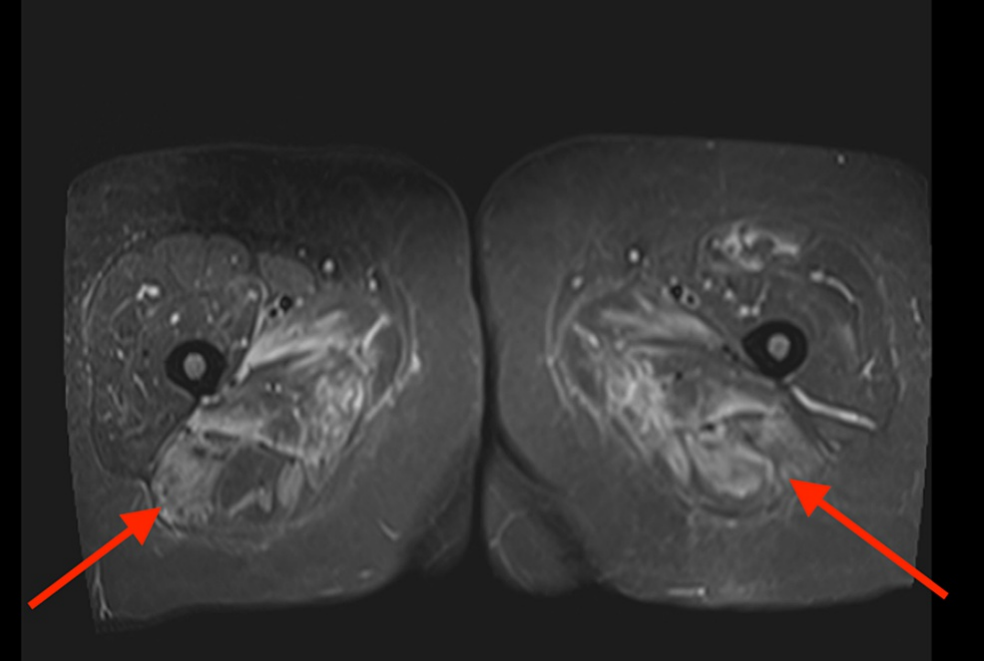
MRI Axial STIR sequence. Edema in bilateral posterior thigh muscles and surrounding fascia. STIR: Short tau inversion recovery.

**Figure 2:**
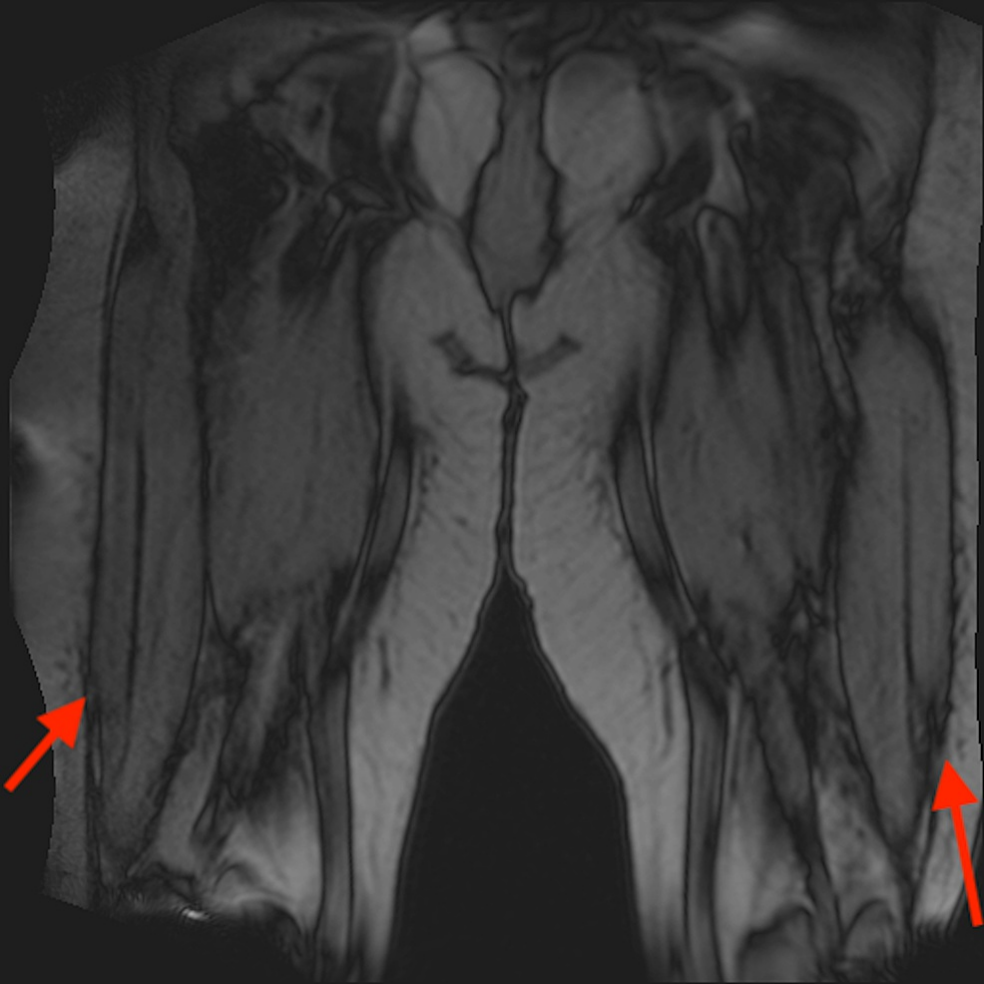
MRI coronal non-fat sat T1. No significant fatty atrophy of posterior thigh muscles.

A 43-year-old Caucasian female initially presented to her primary care physician (PCP) with the chief complaint of progressive weakness of symmetric upper and lower proximal limbs for 4-5 weeks. Symptoms started suddenly and progressively worsened, and she was also experiencing diffuse muscle aches. The patient was having difficulties climbing stairs, standing from a sitting position, and combing her hair. The patient had type 2 diabetes mellitus, hyperlipidemia, and migraine headaches. She had been on atorvastatin, 40 mg daily, for almost five years. Initial outpatient lab work showed an elevated CPK level of 8000 IU/L and the patient was referred to the ED by her PCP for further evaluation. Atorvastatin was discontinued. The patient was started on intravenous hydration for possible rhabdomyolysis. The patient’s symptoms did not improve after aggressive hydration in the hospital, and the CPK level remained persistently elevated above 7000 IU/L. An MRI of proximal lower extremities showed diffuse, symmetric proximal lower extremity muscle edema. MRI also did not show significant fatty atrophy of posterior thigh muscles and this was indicative of acute myopathy (Figures 1 and 2).

Clinical symptoms of significant muscle weakness, very high CPK level, and abnormal MRI findings initially raised suspicion for inflammatory myositis. To rule out inflammatory myositis, a muscle biopsy was performed. The patient was empirically started on 60 mg of prednisone daily for suspected inflammatory myositis pending biopsy result. The muscle biopsy report was suggestive of statin-induced myopathy. Moreover, the subsequent workup showed positive antinuclear antibodies (ANA), negative extractable nuclear antigens (ENAs), and very high HMGCR antibodies (titer > 200). The patient was diagnosed with statin-associated IMNM. She reported resolution of muscle achiness, but no improvement of weakness with corticosteroid treatment. Due to a lack of adequate response to high-dose corticosteroids for eight weeks, she was started on IVIG 2 gram per kilogram of body weight per month for three months. In outpatient follow-up, the patient reported resolution of all muscle symptoms, CPK had been normalized and the patient did not require any further immunosuppressive drugs.

## Discussion

Statins have been reported to be associated with inflammatory myopathies like PM, DM, inclusion body myositis, and IMNM [5]. Statin-associated inflammatory myopathies are characterized by proximal muscle weakness with elevated serum CPK levels [5,6,7]. The patient presented in her PCP's office with progressive proximal muscle weakness and muscle pain with very high CPK. The patient was on 40 mg atorvastatin for five years. Necrotizing myopathies have a similar clinical presentation to other inflammatory myopathies such as PM and DM. Necrotizing myopathy is distinguished from the other inflammatory muscle diseases by the lack of inflammatory infiltrates on muscle biopsy [5]. Moreover, muscle biopsy is not necessary for the diagnosis of IMNM, and it is less specific than immunoassays [9]. In this case, a muscle biopsy was performed, which was suggestive of statin-induced myopathy and ruled out inflammatory myopathies. Statin-induced necrotizing myopathy is distinguished by a specific anti-HMGCR antibody assay. It is not clear if there is a causal relationship between anti-HMGCR antibodies in patients with necrotizing myopathy, but it has been suggested that statins can provoke this syndrome [7]. In our case, the patient had a very high anti-HMGCR antibody (titer >200). Anti-HMGCR antibody also suggests that statins can unmask an immune-mediated myopathy [10]. These autoantibodies continue to persist long after statin discontinuation and result in ongoing tissue damage [11]. The mechanism of statin-induced myopathy is not clearly understood, but it may involve upregulation of major histocompatibility complex (MHC)-I expression and antigen presentation by muscle fibers induced by statins [12]. It has also been suggested that statin may cause the more common toxic myopathy and thereby expose neoantigens to the immune system, which may be responsible for autoimmune myopathy [6].

Statin-induced myalgia is a self-limited condition and the patient's symptoms improve within weeks or months of discontinuing statin [3]. However, statin-associated inflammatory myopathy does not improve after discontinuation of statin and often requires immunosuppressive therapy. Anti-HMGCR myopathy, a variety of statin-associated IMNM is difficult to treat due to continued antibody production long after discontinuation of the offending agent [9]. There are no established guidelines for the management of statin-associated IMNM regarding both therapeutic regimen and duration. Case reports have been published with statin-induced necrotizing myopathies where patients were required multiple immunosuppressive therapies for treatment [5]. However, a case report has been published where monotherapy with IVIG successfully treated statin-associated IMNM [13]. Our patient had very little clinical improvement with a high-dose steroid. Moreover, she was also experiencing the adverse effects of high-dose steroids, such as weight gain and inability to sleep. She was successfully treated with IVIG for three months. During subsequent outpatient follow-up, the patient remained in remission and required no further immunosuppressive therapy.

## Conclusions

Statin-associated IMNM is rare, which does not respond to the discontinuation of statin alone. Lack of improvement in muscle strength and persistently elevated CPK level after statin discontinuation necessitate the importance of further workup including a muscle biopsy. After the diagnosis is confirmed, patients with IMNM often require aggressive immunotherapy. Due to the increasingly widespread use of statins, clinicians should consider the possibility of statin-associated IMNM in patients whose myopathic symptoms and elevated CPK levels persist despite discontinuation of statins. Further studies are needed for a better understanding of the pathogenic mechanisms, risk factors for developing the disease, management, and long-term prognosis of this disease.
